# Consensus on Recommendations for Safe Sexual Activity during the COVID-19 Coronavirus Pandemic

**DOI:** 10.3390/jcm9072297

**Published:** 2020-07-20

**Authors:** Francisco Cabello, Froilán Sánchez, Josep M. Farré, Angel L. Montejo

**Affiliations:** 1 Instituto Andaluz de Sexología y Psicología, Alameda Principal 21, 5º, 29001 Malaga, Spain; fcabello@iasexologia.com; 2 Centro de Salud de Xàtiva, Avenida de Ausìas March s/n. Xàtiva, 46800 Valencia, Spain; consultafsanchez@gmail.com; 3 Department of Psychiatry, Psychology and Psychosomatics, Dexeus University Hospital, Carrer de Sabino Arana, 5, 08028 Barcelona, Spain; jmfarremarti@gmail.com; 4 Hospital Universitario Psychiatry Department, University of Salamanca Nursing School, Institute of Biomedical Research (IBSAL). Av., Donantes de Sangre SN, 37007 Salamanca, Spain

**Keywords:** sexual activity, COVID-19, SARS-CoV-2, recommendations, consensus, sexual risk

## Abstract

Sexual activity offers numerous advantages for physical and mental health but maintains inherent risks in a pandemic situation, such as the current one caused by SARS-CoV-2. A group of experts from the Spanish Association of Sexuality and Mental Health (AESexSAME) has reached a consensus on recommendations to maintain lower-risk sexual activity, depending on one’s clinical and partner situations, based on the current knowledge of SARS-CoV-2. Different situations are included in the recommendations: a sexual partner passing quarantine without any symptoms, a sexual partner that has not passed quarantine, a sexual partner with some suspicious symptoms of COVID-19, a positive sexual partner with COVID-19, a pregnant sexual partner, a health professional partner in contact with COVID-19 patients, and people without a sexual partner. The main recommendations include returning to engaging in safe sex after quarantine is over (28 days based on the duration one can carry SARS-CoV-2, or 33 days for those who are >60 years old) and all parties are asymptomatic. In all other cases (for those under quarantine, those with some clinical symptoms, health professionals in contact with COVID-19 patients, and during pregnancy), abstaining from coital/oral/anal sex, substituting it with masturbatory or virtual sexual activity to provide maximum protection from the contagion, and increasing the benefits inherent to sexual activity are recommended. For persons without a partner, not initiating sexual activity with a sporadic partner is strongly recommended.

## 1. Introduction

Sexuality is one of the aspects of personality in which the degree of intimacy and privacy is great. Asking patients about their sex life often arouses misgivings and feelings of shame and/or guilt [1]. However, scientific evidence shows that successful sexuality benefits males and females physically and emotionally to, having a favourable impact on their quality of life. There is evidence that sexual activity has advantages for humans, including increasing our longevity [2,3,4] and improving our immune system, among others [5,6]. Additionally, successful sexual activity increases psychic wellbeing by improving mood, even in depressed and high-anxiety patients [7,8], falling asleep [9]; stress [10]; relaxation [11]; physical form and providing a younger body image [12] thereby contributing to the prevention of post-traumatic stress and anxiety disorders [13]. Sexual experience regularises the menstrual cycle [14], relieves dysmenorrhea and reduces the risk of endometriosis [15]. Sexual dysfunctions can cause some interpersonal conflicts by deteriorating either self-esteem or partner relationships [16]. Additionally, it may constitute an early sign of some organic pathology such as cardiovascular [17], neurological or endocrine diseases. There is also some evidence that sexual inactivity correlates with an increased frequency of cancer, need for major surgery, worsening mental health and the increase of cognitive decline and cardiovascular disease risk factors such as diabetes, hypertension and hypercholesterolemia [18]. 

The effects of SARS-CoV-2 on human sexual and reproductive function, including whether the virus passes the blood–testis and ovary barriers and whether there is any effect on sexual hormone production, are still unknown [19]. Additionally, some studies are currently seeking to identify similar impacts across the different populations impacted by HIV [20].

In the current SARS-CoV-2 pandemic situation, sexual activity during quarantine could be a relevant aid in reducing the onset of post-traumatic stress and anxiety disorders that were experienced in other previous pandemic confinements. In Canada, during the outbreak of Severe Acute Respiratory Syndrome (SARS) in 2003, a high level of acute stress was observed among health workers [21]. Ebola virus confinement in Africa also increased suicides [22] and gender-based violence [23]. During the Australian equine fever quarantine, a high level of anxiety was observed in 34% of those confined compared to 12% in those not confined [24]. In China, some of the health workers quarantined during the SARS epidemic-maintained symptoms of post-traumatic stress disorder three years later [25].

Sexual satisfaction is a good predictor of global life satisfaction in young people and older adults [26]. In a large survey on sexual health in Spain [27], people were interviewed about their motivation for sexual intercourse, and the vast majority pointed out that the main reason was either the search for emotional intimacy or to satisfy the need to love and be loved. Additionally, sexuality, as a basic aspect of mental health, is a current topic of interest for clinicians and researchers [28]. 

There is some literature indicating the potential benefits of increased sexual activity during periods of forced isolation indicating that those who maintain frequent in-person, but not remote, social and sexual connections have better mental health outcomes [29]. Given the psychologically negative repercussions of previous quarantines and the preventive benefits of healthy sexuality, it is reasonable to maintain one’s safe sexual frequency. However, sexual intercourse requires close physical contact, and SARS-CoV-2 is very easily transmitted with this level of closeness [30]. Physical contact entails high viral exposure. When sharing a home with a COVID-19-positive person, the virus has been detected in 63.2% of room air samples and 66.7% of corridor air samples [31].

Other known coronaviruses do not appear to be sexually transmitted, but SARS-CoV-2 has been found in bodily fluids such as the saliva, mucus, and faeces of infected people, albeit slightly less in urine (6.9%). Some recent studies have reported the virus to be present in the testicular seminal duct [32,33] compromising the safety of sexual intercourse by persistence for at least 2 weeks postinfection in urine, faeces and nasopharynx secretions. Considering that 80% of those infected have mild symptoms or are asymptomatic, it is advisable to take some precautions at least during quarantine. The use of condoms and noncoital behaviour that does not involve direct contact with semen is highly recommended [34,35].

The virus was very recently found in the vaginal discharge of an infected 65-year-old female even while she was receiving oral lopinavir/ritonavir plus remdesevir. After two previous negative results, the vaginal swab tested positive via a real time reverse transcriptase-polymerase chain reaction on days 7 and 20 from symptom onset [36]. This new finding raises the possibility that sexual intercourse could be an additional direct vector of infection, adding to the recent evidence of a likely faecal–oral transmission vector [37], or indirectly by exposure of the rectal mucosa to saliva [38]. Additionally, patients can persistently test positive on rectal swabs even after negative results from nasopharyngeal testing [39]. Thus sexual, transmission may be possible despite apparent clinical recovery. Using real-time reverse transcription polymerase chain reaction to routinely test for SARS-CoV-2 in faeces was recently recommended [37]. Patients’ sexual habits should be routinely investigated in order to avoid direct sexual practices if infected with COVID-19. Physicians should always address these questions in epidemiologic surveys on transmission routes in order to determine effective strategies to control infection.

Information about changes in sexual habits in the isolated population and in those infected by the virus is scarce so far. An increase in both, sexual desire and the frequency of sexual intercourse during the current COVID-19 pandemic, compared to the previous 6–12-month period, has recently been shown, although the quality of sex decreased significantly [40]. However, another recent study showed that during the COVID-19 outbreak, the frequency of sexual activity in China decreased significantly in men and women, accompanied by a lowering of risky sexual behaviours [41].

The main objective is to avoid contagion by COVID-19 and, at the same time, maintain, as far as possible, active sexuality, given the multiple advantages that healthy sexuality brings according to scientific evidence.

## 2. Method

Due to the ease of contagion and the lack of information about the possible transmission of SARS-CoV-2, a group of experts from the Spanish Association for Sexuality and Mental Health, covering the fields of sexology, psychiatry, psychology and medicine reached a consensus. The multidisciplinary panel included four experts in the fields of family medicine, sexology, epidemiology, psychology and psychiatry. A bibliographic search was performed in the Medline, Scopus, PsycInfo and Web of Science databases without time limits. After searching the information sources, two reviewers independently preselected potentially relevant references using the keywords; sexual * AND coronavirus OR COVID- 19 OR pandemic (645 refs). After preselection the search was refined, duplicates were removed, and 83 refs were found. After reading the complete articles, those that would ultimately form part of the review were selected (38). Based on the current knowledge of the scientific literature, and considering the absence of either clinical guidelines or recommendations in this regard, the authors have developed a consensus on some specific recommendations to maintain safe sexual activity and to prevent the transmission of COVID-19. The authors carried out three preliminary drafts until a full consensus of the final text was reached.

## 3. Clinical Recommendations for Safe Sexual Activity

In order to avoid the risk of contagion, the main recommendation is that tongue kissing and oral-sex relationships should be avoided. As indicated by recent recommendations from the New York Department of Health, “You are your safest sexual partner” [42]. Thus, during the pandemic, and until the end of quarantine, it is a good time to devote oneself to autoerotic growth, which means, improving sexual health, and therefore mood, by training to optimise sexual response.

Under confinement with a sexual partner (not always a household partner), including the full diversity of partner types (homo, hetero, bisexual, nonheteronormative couples with polyamory or those who maintain living apart), there are several possibilities we can recommend to improve safe sexual activities by avoiding the risk of contagion to the greatest extent. To clarify the concept of “safe quarantine”, our recommendation includes avoiding contact with high-risk populations during quarantine and avoiding restarting sex when in contact with a confirmed or highly suspected case. We contemplate two main scenarios: (a) partners living in the same household and (b) partner/s not living in the same household/starting a new relationship or polyamory.

- Partner/s living in the same household.
  1. A sexual partner after passing complete asymptomatic quarantine.
  2. A sexual partner during quarantine.
  3. A sexual partner with suspicious symptoms of COVID-19.
  4. A sexual partner that is positive for COVID-19
  5. A pregnant sexual partner.
  6. A sexual partner in contact with COVID-19 patients.
- Partner/s not living in the same household/starting a new relationship or polyamory.

### 3.1. Partner/s Living in the Same Household

#### 3.1.1. A Sexual Partner after Passing Complete Asymptomatic Quarantine

Current information indicates that the average incubation period for COVID-19 is 5.1 days (95% CI 4.5–5.8 days) and that 97.5% of cases developed symptoms in 11.5 days (95% CI: 8.2–15.6 days). The incubation period before the onset of fever is 5.7 days, and 98% of those infected have a fever in the first 12.5 days. Only 1% develop symptoms after 14 days; there is a small but real possibility of symptomatology at 28 days after infection [43].

The recommendation is that after 28 days of confinement without symptoms and without external contact, sexual intercourse can be carried out according to the usual habits of the couple, but while increasing hygiene before and after sexual activity. This would be a good time to enhance sexual creativity by increasing communication about one’s preferences and desirable experiences, possibly by sharing fantasies and testing new scenarios, engaging in erotic readings, or viewing erotic films.

Before starting sexual activity, proper handwashing becomes essential to avoid virus transmission through fomites [44] and by touching the T-zones (mouth, nose and eyes) of the sexual partner’s face [45]. It is also advisable to recommend strategies to avoid self-contact with one’s own T-zones, as it happens without realisation at an average of 23 times per hour [46].

#### 3.1.2. A Sexual Partner during Quarantine (before 28 Days)

In this scenario, we must take into account that the severity of COVID-19 depends on several factors, including viral load. It is possible that one member of the couple could be contaminated but asymptomatic as seen in a sample of 157 cases in Singapore where 6.4% of presymptomatic transmission was identified [47]. In this case, sexual intercourse could be a major risk of contagion. The recommendation, before 28 days, is that one can practice safe sex using penetrative positions from behind, avoiding kissing, oral and anal sex, and by always using condoms.

#### 3.1.3. A Sexual Partner with Suspicious Symptoms of COVID-19

If a partner shows any suspicious symptoms, such as fever (which may be intermittent), cough, diarrhoea, severe and unexplained tiredness, sore throat, anosmia, hypogeusia, or other symptoms associated with COVID-19, he or she may be in the incubation period or suffering from a mild form of the disease. The recommendation would be to avoid direct sexual activity. As a substitute, performing self-stimulation (masturbation while simultaneously keeping the safety distance of approximately 2 m), narrating erotic fantasies, sharing visualisations of erotic scenes, or using erotic board games might be suggested.

#### 3.1.4. A Sexual Partner Positive for COVID-19

If a sexual partner has tested positive for SARS-CoV-2, having sex is a possible source of contagion, so it is advisable to isolate in separate rooms. However, it is advisable to continue to practice some kind of sexual activity while avoiding physical contact, as recommended previously. One can, for example, use telematic applications to maintain erotic activities. It should be considered that people with active COVID-19 infections may not want to engage in any sexual activity (even remote/virtual connections,) depending on the severity of their symptoms and personal preferences. Once symptoms disappear, there is a high probability of contagion, as the virus persists in nasal secretions for an average of 9.5 days and in stool from 11 to 16 days (20 days if treated with corticosteroids) [48]. The duration for carrying SARS-CoV-2 in COVID-19 (between 16–28 days) can increase up to 33 days in those over 60 years old, as well as in very severe cases [49]. Waiting for this period of time before having coital sex and maintaining the recommended precautions for confined stable sex partners without passing quarantine are recommended. 

#### 3.1.5. A Pregnant Sexual Partner

It is currently unknown whether pregnant individuals are more likely to get sick with COVID-19, and there is still little information on the severity of the disease [50,51]. Other coronaviruses and viral respiratory infections, such as the flu, may carry a higher risk of developing complications or serious illnesses in pregnant women. Therefore, since this is a population at risk, precautions should be increased [52]. Pregnant individuals do not appear to be at greater risk than the general population, except for the presence of other associated factors, such as preeclampsia, hypertension, diabetes, and uterine atony. These individuals should follow the same preventative measures as those indicated for the general population. Fetal transmission is unlikely as no viruses have been reported in either the umbilical cord, amniotic fluid, breast milk, or neonatal pharynx smears. However, in addition to the usual records, fetal ultrasounds and cardio recordings are recommended. In the case of maternal infection, there is a risk of postpartum transmission, for which screening tests will be needed [53,54]. However, a recent joint review, including 37 infected pregnant women and 38 newborns in Iran/China/USA aged 23–40 years-old, a placental transmission during the pregnancy was observed, while keeping normal amniotic fluid, vaginal discharge and milk secretion. No teratogenic effects were shown. However, the risk of infection with breast milk, cough or other vectors was high for 5–7 days after birth, so it is advisable to avoid breastfeeding if the mother is infected [55].

The recommendation is to use the same identified sexual strategies for stable sexual partners confined without passing quarantine, thus avoiding kissing, oral sex, and anal penetration, and using “a tergo” and posterior positions.

#### 3.1.6. A Sexual Partner in Contact with COVID-19 Patients

The health workers, caregivers, and professionals in contact with infected people in hospitals, residential centres, or similar areas could spend a great deal of time exposed to the virus, possibly without adequate protection. Thus, there is a high risk of accumulating a large viral load. The recommendation would be to follow the same strategy used for contaminated people (i.e., virtual sex).

### 3.2. Partner/s Not Living in the Same Household/Starting a New Relationship or Polyamory

Under no circumstances should sex be performed in vivo with a new partner unless there is the certainty that the partner has been immunised against the virus. For those who do not have a partner and for very erotophilic people, nonheteronormative couples with polyamory or those who maintain living apart, the recommendation is abstinence from sexual intercourse until the incubation time passes without symptoms after starting the coexistence. Additionally, it might be worth using sexting or virtual sex. Some swingers clubs organise erotic encounters through platforms in order to have virtual group sex. The measures that are generally taken to contain SARS-CoV-2 have undoubtedly decreased the professional activities of sex workers, and fewer casual sexual encounters in this context may affect individual risks for HIV infection and other STIs [56]. In order to minimise the risk of infection when sporadic sexual intercourse occurs, condoms, dental dams or similar protection methods should be applied.

### 3.3. Summary of Recommendations

In summary, we recommend maintaining an adequate level of eroticism and sexual activity, both in confinement and throughout the pandemic. This, along with other factors such as physical activity, social distancing or smoking cessation, will allow the minimisation of some part of its negative effects. However, it is necessary to meet the standards established by international organisations for the prevention of Covid-19 [57], given that sexual relations, regardless of sexual orientation—heterosexual, homosexual or bisexual—can facilitate its spread. A summary of the recommendations can be seen in Table 1, considering the previous different scenarios contemplated.

## 4. Discussion

Our recommendations are comparable to those made by institutions such as New York Citi Health [53], but so far, no specific recommendations have been published on safe sexual practices to avoid the transmission of the virus by other sources of trusted information such as the Centers for Disease Control and Prevention (CDC) or the World Health Organization.

There is certainty that the vast majority of the global population will survive this pandemic, but associated stressors, such as uncertainty about the end of the pandemic, fear of contagion, and economic and social impairment, facilitate a weakening of the immune system [58,59]. As there is evidence that sexuality promotes the immune system and physical and mental health, it is advisable to maintain an acceptable degree of sexual intercourse. There is some evidence that healthy sexual activity improves the immune system and serves as a more preventive factor against SARS-CoV-2. On the other hand, healthy, safe and frequent sexual activity might be attenuating (even if there is no evidence yet) the negative psychological effects associated with the infection. Unfortunately, at this moment, there are no vaccines available for SARS-CoV-2 and we do not yet know how durable immunity is after an infection. Currently, the epidemic is still spreading, and there are no effective means to prevent the infection. As most vaccines are under design and preparation, there is currently no possibility of having “certainty” around immunisation status [60].

Additionally, SARS-CoV-2 may cause neurologic and psychiatric effects such as delirium in some patients in the acute stage and depression, anxiety and post-traumatic stress disorder in the long term [61]. Therefore, the protective benefits of sexual activity and the maintenance of a suitable couple relationship could be beneficial to avoid the psychiatric and psychological deterioration that is secondary to the pandemic. 

On the other hand, it is very relevant not to stigmatise healthcare workers and consider them contaminated people. However, if the risk is high due to being in permanent contact with COVID-19 patients, precautions should be taken to avoid infections due to the existence of frequent asymptomatic cases. In this sense, pregnant women should not be stigmatised either, although additional precautions are necessary to avoid possible consequences after infection in the mother and the fetus.

Undoubtedly, the pandemic has implied some big changes in the sexual behaviour of special groups such as sex workers, where it is difficult to issue recommendations because it is their only livelihood in low-income countries. The recommendation of sexual abstinence in this group is aimed at promoting changes to sex work due to increased potential exposure to infection and various health concerns, however, they could be very difficult to apply. Innovative strategies launched by the leaders of sex workers and health workers, implementing new health protection guidelines and clinical spaces, are especially instructive [62]. Additionally, other vulnerable populations such as men who have sex with men (MSM), require special attention. A recent survey in the USA on sexual habits in MSM during the pandemic showed that half had fewer sex partners and most had no change in condom access or use with increased difficulties in accessing HIV testing, prevention and treatment services. In addition to our recommendations to reduce the risk of sexual contact, it is necessary to apply other strategies such as telehealth and mailed testing and prevention supplies to avoid increased HIV incidence among MSM during the COVID-19 pandemic [63].

The risk of unwanted pregnancies is a consequence of the COVID-19 pandemic [64], because sexual and reproductive health services have remained closed during the pandemic. Fortunately, in some countries, remote resources have allowed them to continue their preventive function by prescribing contraceptive methods, but unfortunately, in many others it has not been possible. We consider both, sexual and reproductive healthcare to be a priority, guaranteeing access to contraception as well as adequate information on sexual and reproductive health.

Some sexual intercourse-related scenarios may not be covered by these recommendations, and additionally, there is a lack of full information on virus behaviour and its impact on the sex life of infected patients and asymptomatic transmitters. However, keeping sex life healthy and safe is one of the biggest challenges for health prescribers and for the general population in this pandemic. Furthermore, the peculiarities of sexual minorities during the pandemic are not yet known so these recommendations may not be widespread. There is not enough information about how the treatments received by infected patients affect their sex life or whether there are modifications to desire, arousal or orgasm compared to the previous stage. Although many uncertainties still remain, the study of the sex life of this population could clear up scarce information about how the virus affects both the sexual and reproductive life. However, the benefits of maintaining a healthy sex life, harnessing its qualities and preventing contagion should be a priority in all sexual activity scenarios.

## 5. Limitations

These recommendations do not cover all minority sexual behaviours due to the great heterogeneity of human sexuality. However, they include most diversity of sexual activity regardless of orientation: heterosexual, homosexual or bisexual. Our proposals for confinement include maintaining a security period of 28 days, before the couple can restart their regular sexual practices, since 1% of persons affected by Covid-19 develop symptoms after an incubation period of 14 days. Therefore, although we know that this could be a difficult recommendation for many couples to accept, we consider it relevant to safeguard security as much as possible. Another relevant limitation is related to some still unknown facts, such as the duration of the immunity in people who have overcome the disease. This would be crucial to avoid reinfections when patients are supposedly immunised but do not respect the preventive measures implemented.

## 6. Need for Research

Further research is needed to obtain more precise data on the pathophysiology, prevention and treatment of COVID-19, as well as to better understand the duration and level of immunity that overcoming the disease confers. With more scientific evidence, these are transcendental factors when recommending actions, that could avoid contagion through sexual activity. Progress in the understanding of these aspects, through well-designed observational, case-control and experimental studies, will allow us to modify and enrich our current recommendations.

## 7. Conclusions

Staying at home and complying with strict confinement standards is the most active way to fight SARS-CoV-2. Strengthening the immune system along with other factors such as increased positive interpersonal relationships and rewarding occupational activities, will help us fight the negative effects of the pandemic. This must be done without forgetting to reserve time for erotic activity that fosters creativity and communication.

Finally, confinement protects us from the risk of contagion, but at the same time, it can be an opportunity for the cultivation of eroticism through communication, fantasies, and exploration of a new self and heteroerotic scenarios, taking into account the health precautions established by international organisations against COVID-19.

## Figures and Tables

**Table 1:** Summary of recommendations for sexual activity during the COVID-19 pandemic.

A. Partner/s Living in the Same Household	Symptoms	Recommendation
1. After passing quarantine (28 days)	NO	Have regular sex. Increase hygiene measures. Encourage communication and sexual creativity.
2. During quarantine	NO	Sexual activity with a condom and with posterior coital penetrative positions. Increase hygiene measures. Avoid kisses, oral, and anal sex. Encourage communication and sexual creativity
3. With any suspicious clinic at any time	Any suspected symptoms of COVID-19	Avoid coital sexual activity for 28 days. Increase hygiene measures. Self-stimulation (masturbation while simultaneously keeping the safety distance of approximately 2 m), narrate erotic fantasies, share visualizations of erotic scenes, use erotic board games. Encourage communication and sexual creativity.
4. With positive COVID 19	With/without fever	Avoid coital sexual activity for 28 days (33 in >60 y.o.) Isolation in a separate room. Virtual sex with self-stimulation, narrate erotic fantasies, share visualizations of erotic scenes, etc. Encourage communication and sexual creativity.
5. Pregnant	NO	Sexual activity with posterior penetrative positions. Increase hygiene measures. Avoid kissing, oral, and anal sex. Use condoms. Encourage communication and sexual creativity.
6. In contact with COVID-19 patients	NO	Avoid coital sexual activity. Virtual sex with self-stimulation, narrate erotic fantasies, share visualizations of erotic scenes, etc. Encouraging communication and sexual creativity.
B. Partner/s not living in the same household/starting a new relationship or polyamory.	NO	Avoid sporadic couples. Virtual sex activity. Increase hygiene measures. Use of condom, dental dam or similar protection methods.

y.o. = years old
